# Translational Insights in the Landscape of Salivary Gland Cancers: Ready for a New Era?

**DOI:** 10.3390/cancers16050970

**Published:** 2024-02-28

**Authors:** Francesco Perri, Roberta Fusco, Francesco Sabbatino, Morena Fasano, Alessandro Ottaiano, Marco Cascella, Maria Luisa Marciano, Monica Pontone, Giovanni Salzano, Maria Elena Maiello, Massimo Montano, Ester Calogero, Roberta D’Aniello, Piera Maiolino, Fortunato Ciardiello, Alessia Zotta, Salvatore Alfieri, Franco Ionna

**Affiliations:** 1 Istituto Nazionale Tumori di Napoli, IRCCS “G. Pascale”, Via Mariano Semmola, 80131 Napoli, Italy; a.ottaiano@istitutotumori.na.it (A.O.); mcascella@unisa.it (M.C.); m.pontone@istitutotumori.na.it (M.P.); mariaelena.maiello@istitutotumori.na.it (M.E.M.); m.montano@istitutotumori.na.it (M.M.); ester.calogero@istitutotumori.na.it (E.C.); r.daniello@istitutotumori.na.it (R.D.); p.maiolino@istitutotumori.na.it (P.M.); f.ionna@istitutotumori.na.it (F.I.); 2 Medical Oncology Devision, IGEA S.p.A., 80013 Naples, Italy; r.fusco@igeamedical.com; 3 Medical Oncology Department, Università degli Studi di Salerno, Via Giovanni Paolo II, 132, 84084 Salerno, Italy; fsabbatino@unisa.it; 4 Department of Precision Medicine, Università degli Studi della Campania Luigi Vanvitelli, 80128 Naples, Italy; morena.fasano@unicampania.it (M.F.); fortunato.ciardiello@unicampania.it (F.C.); alessia.zotta@studenti.unicampania.it (A.Z.); 5 Department of Neurosciences, Reproductive and Odontostomatological Sciences, University of Naples Federico II, 80131 Naples, Italy; giovanni.salzano2@unina.it; 6 Head and Neck Medical Oncology Department, Fondazione IRCCS Istituto Nazionale dei Tumori, 20133 Milan, Italy; salvatore.alfieri@istitutotumori.mi.it

**Keywords:** salivary glands carcinoma, targeted therapy, driver mutations, oncogenes, translational research

## Abstract

**Simple Summary:**

In this review, we will analyze the main mutations predominant in the various histotypes of salivary gland carcinomas, we will discover whether they behave as driver mutations, and finally we will make hypotheses relating to the best targeted therapy to be used in salivary gland carcinomas, apart from polychemotherapy.

**Abstract:**

Salivary gland carcinomas (SGCs) are rare neoplasms, representing less than 10% of all head and neck tumors, but they are extremely heterogeneous from the histological point of view, their clinical behavior, and their genetics. The guidelines regarding their treatment include surgery in most cases, which can also play an important role in oligometastatic disease. Where surgery cannot be used, systemic therapy comes into play. Systemic therapy for many years has been represented by polychemotherapy, but recently, with the affirmation of translational research, it can also count on targeted therapy, at least in some subtypes of SGCs. Interestingly, in some SGC histotypes, predominant mutations have been identified, which in some cases behave as “driver mutations”, namely mutations capable of governing the carcinogenesis process. Targeting these driver mutations may be an effective therapeutic strategy. Nonetheless, it is not always possible to have drugs suitable for targeting driver mutations—and targeting driver mutations is not always accompanied by a clinical benefit. In this review, we will analyze the main mutations predominant in the various histotypes of SGCs.

## 1. Background

Salivary gland carcinomas (SGCs) are rare tumors arising from major and minor salivary glands. The prevalence of SGCs is less than 1%, representing about 5–8% of all head and neck tumors. SGCs have a multifactorial etiology including ionizing radiation, chemical exposure, obesity, autoimmune disorders, and viral infections. SGCs can be divided into more than 20 different histotypes, based on their histology. Among them, the most frequent ones are: Adenoid Cystic Carcinoma (AdCC), Salivary Duct Carcinoma (SDC), Muco-Epidermoid Carcinoma (MEC), Acinic Cells Carcinoma (AciCC), Secretory Carcinoma (SC), Adenocarcinoma not Otherwise Specified (NOS), and Carcinoma ex Adenoma Pleomorphic (CAP) [1,2]. Among the various histological types, MEC is the most common primary salivary gland malignancy, followed by AdCC, SDC, and AciCC.

Surgery represents the backbone of treatment for early stage, locally advanced and selected cases of recurrent/metastatic disease. Radiation therapy is mostly performed with adjuvant intent following radical surgery although in selected cases concurrent chemo-radiation (e.g., cisplatin plus radiotherapy) may be employed with radical intent [3,4,5]. Systemic therapy represents standard of care for recurrent/metastatic disease, mainly in those cases where concomitant surgery of primary tumors and metastatic sites cannot be performed. Polychemotherapy is the therapeutic standard in all histotypes of SGCs and the most commonly used regimens are those containing platinum salts (i.e., cisplatin and carboplatin) including Carboplatin–Taxol, Cisplatin–Adriamycin–Cyclophosphamide (PAC), Cisplatin–Vinorelbine, Cisplatin–5Fluorouracil, and Cisplatin–Doxorubicin. Response rate obtained from polychemotherapy is widely variable, depending by the histotype, as well as by the aggressiveness of the tumor. Globally, the Overall Response Rate (ORR, as the sum of complete and partial response) ranges from 20 to 40% [6,7,8,9,10].

Some SGCs are characterized by so-called “driver mutations”, namely genetic changes capable of promoting and driving carcinogenesis. Over-expression of the androgen receptor (AR) and HER2 amplification have been frequently reported in SDCs. In addition, AR antagonists, as well as HER2-specific monoclonal antibodies, have been reported for favorably treating AR or HER2 over-expressing SDCs [11].

**Figure 1:**
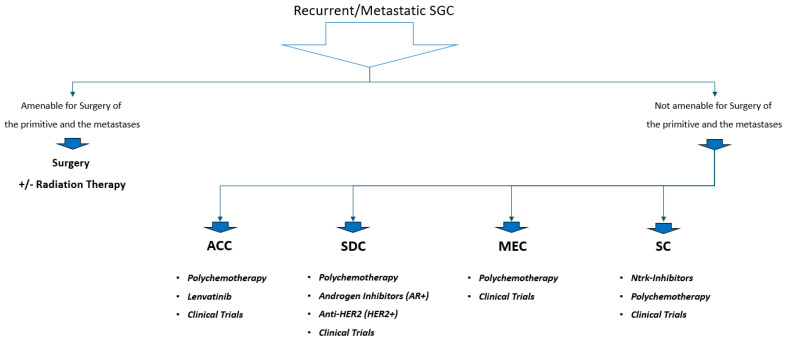
Standard therapy option for recurrent/metastatic SGC. Note—SGC: Salivary Gland Carcinoma; RT: Radiation Therapy; ACC: Adenoid Cystic Carcinoma; SDC: Salivary Duct Carcinoma; MEC: Muco Epidermoid Carcinoma; SC: Secretory Carcinoma.

However, specific molecular target therapy is currently used in AdCC, and the drug used in clinical practice is Lenvatinib, which is a broad-spectrum tyrosine kinase inhibitor (TKI) capable of mainly blocking VEGFR [12]. Figure 1 shows the standard therapy options for recurrent/metastatic SGCs.

Translational research may be defined as the rapid application of the results obtained from basic research into clinical practice. The methodology used by translational research often involves the analysis of some genetic or molecular alterations identified with a certain frequency in solid tumors, starting from the assumption that such alterations can be promoters of carcinogenesis. Whenever possible, the aim is to design a drug (or use one already available) to target the aforementioned alterations. The comprehensive study of the genetics of SGCs has identified several mutations with a high frequency particularly histotypes of SGCs. In some cases, they could behave like “driver mutations”. Therefore, the aim of this review will be to analyze the genetics of SGCs and overview targetable genetic abnormalities. This need arises from the evidence that finding (identifying) the so-called “weak point” of tumors, namely the targetable genetic abnormality, is the primary objective of translational research, which is one of the most important weapons to solve/address the clinical unmet needs.

In this review, we did chose to treat some particular histotypes because they are the most frequently encountered in the clinical practice, and also because they are those in which targetable alterations have been yet found.

## 2. Adenoid Cystic Carcinoma (AdCC)

Adenoid cystic carcinomas are often found in minor salivary glands, especially in the hard palate. AdCCs are relatively rare types of cancer, but they also are the second most frequent subtype of salivary glands cancer, accounting for 10% of all salivary gland tumors. AdCC is characterized by a high capacity to locally recur and displays a poor prognosis with a survival rate of 24–45% at 5 years [13]. Lung metastases and perineural invasion in the primary site are very frequent. Surgery, followed by radiation therapy, represents the only valid therapeutic option in both local and locoregional disease. Even in presence of metastatic disease, resection of both locoregional disease and metastatic sites can be an efficient therapeutic approach especially in cases of oligometastatic and indolent disease [14].

In contrast, in advanced recurrent and/or metastatic rapidly progressive disease, as well as in presence of a high number of metastatic lesions, systemic therapy is the gold standard. A wide variety of antiblastic regimens have been employed including cisplatin monotherapy; cisplatin plus anthracycline; cisplatin plus gemcitabine; CAP (cyclophosphamide, doxorubicin, and cisplatin); mitoxantrone; vinorelbine; and epirubicin. Nevertheless, the response rate is low, ranging from 24 to 30%. The most employed scheme is CAP, which can provide an ORR of 25% and median OS of 23.4 months [15,16].

**Figure 2:**
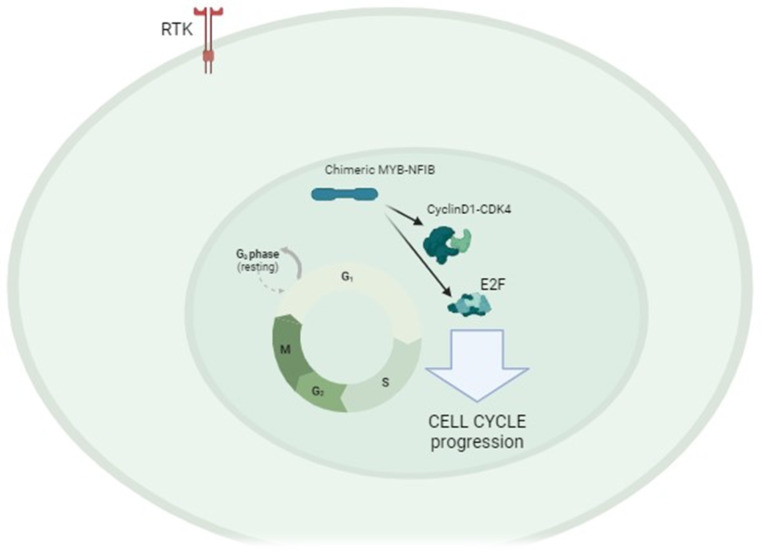
Chimeric enzyme (NFIB-MYB is able to activate some important factors strongly involved in the cell cycle progression, such as Cyclin D1 and E2F. Cell cycle dysregulation is supposed to be the main mechanism of carcinogenesis in AdCC. RTK: receptor tyrosine kinase.

From a genetic point of view, some considerations regarding ACC can be made. First, Tumor Mutational Burden (TMB) [17] is defined as the number of non-inherited mutations per million bases (Mb) of investigated genomic sequence. TMB is reported to be low in AdCC and the most frequent DNA-mutations identified are found in NOTCH1 (25%), KDM6A, CREBP, and SMARCA2 (15%) genes. In addition, 60–80% of cases display a chromosomal translocation t(6;9) which leads to MYB-NFIB chimeric fusion, and over-expression of MYB-oncoprotein. In humans, the gene MYB encodes for the transcription factors named MYB1 and MYB2 [18,19,20]. They can enhance the transcription of several downstream genes, most of them involved in the cell-cycle progression, although they have different targets. Genes regulated by MYB include proteins able to accelerate the cell cycle such as CCNA1, CCNB1, CCNE1, and CCND1, as well as others involved in inhibiting apoptosis induction such as API5, BCL2, BIRC3. MYB is also able to increase the transcription of genes involved in angiogenesis, such as FGF2, KIT, MYC, VEGFA, and cell migration (ICAM1, MMP7, and MMP9) [21]. Figure 2 shows the intracellular pathway stimulated by MYB-NFIB chimeric enzyme, illustrating some of the possible ways to promote carcinogenesis.

NOTCH signaling is a highly conserved pathway in evolutionarily terms. NOTCH receptors undergo a process of cleavage and it is translocated into the nucleus to regulate the transcription of target genes. NOTCH signaling strongly participates to the development and differentiation of cells in multiple tissues and organs. The binding of ligands to extracellular domains of NOTCH allows to initiate its endocytosis, with this latter process inducing receptors to change their conformation, exposing the enzymatic site for its cleavage. The multiple cleavage change NOTCH into the effector form: NOTCH intracellular domain (NICD), which is in turn translocated into the nucleus and functions as a transcription factor. The enzyme responsible for cleavage of NOTCH is the γ-secretase, which are the target for a drugs’ class: the gamma-secretases inhibitors. NOTCH mutations can stimulate cancerogenesis in several ways, but in the AdCC development, it seems that NOTCH mutations are able to increase MYB expression, leading so to tumorigenesis [22].

From the therapeutic point of view, the selective targeting of MYB-oncoprotein has been proposed in several scientific reports. Retinoic Acid (ATRA) has been employed in a phase II trial enrolling 18 patients with advanced chemo-refractory AdCC, demonstrating Stable Disease (SD) as the best response in 61% of patients, for a median duration of 3.7 months [23]. Another way to target MYB is to target its specific transcription targets. A phase II clinical trial of amivantamab, which is a bispecific inhibitor of the MYB transcription target MET and EGFR, is currently ongoing [24].

Targeting NOTCH signaling may be an alternative way to block MYB pathway. Ferrarotto et al. published the results of a phase II trial assessing the role of AL101, a selective gamma-secretase inhibitor, in patients with advanced AdCC harboring activating NOTCH mutations. A total of 77 patients were treated and the Disease Control Rate was 68.7%, albeit with significant gastrointestinal toxicity and asthenia [25].

Brontictuzumab is a monoclonal antibody that selectively targets NOTCH. Ferrarotto et al. published results of a phase I trial enrolling patients with diagnosis of different refractory solid tumors over-expressing NOTCH. Among the 48 patients enrolled in total, 17 had a diagnosis of ACC. Interestingly, ORR in these latter patients was interesting reaching 12%, with a DCR of 25% [26].

Interestingly, almost 80% of AdCC overexpress PSMA (prostate-specific membrane antigen), a transmembrane glycoprotein first studied as an imaging and therapeutic target in prostate cancer. This feature has prompted further investigation of PSMA in salivary gland malignancies, and in particular, therapies aimed to selectively target PSMA have been taken into account [27]. Nevertheless, the preliminary results of a phase I trial analyzing PSMA inhibitors in SGC have been shown at ASCO 2023 and they did not seem promising [28].

AdCCs have also a high c-Kit expression of about 90%. C-kit is a member of the family of receptor tyrosine kinases, and it binds to its physiological ligand, stem cell factor (SCF), also known as c-Kit ligand, leading to several physiological functions. Downstream pathways stimulated by c-Kit activation are PI3K-Akt, RAF/MEK/ERK, JAK/STAT and Src. Activation of c-Kit induces cell proliferation and angiogenesis [29]. Imatinib is an inhibitor of c-Kit, bcr-abl, and PDGFRA (platelet-derived growth factor receptor alpha), and it was used in a number of phase II studies enrolling patients with ACC. Unfortunately, the low obtained ORR did not prompt further evaluations in ACC [30,31,32,33,34,35].

VEGFR (vascular endothelial growth factor) is over expressed in almost 80% of ACC, and this finding paved the way to several clinical trials employing drugs that target angiogenesis. Dovitinib, axitinib, sunitinib, regorafenib, sorafenib, nintedanib, and lenvatinib are drugs mainly targeting VEGFR and PDGFR, and they have been tested in phase II clinical trials enrolling patients with advanced chemo-refractory ACC (Table 1). ORRs ranged from 8 to 16% and PFS values were between 4.3 to 17.5 months. Sorafenib and lenvatinib show the highest ORRs for all tyrosine kinase inhibitors (sorafenib 11% and 16% respectively), and Lenvatinib also demonstrated the best PFS (17.5 months) [32,33,34,36,37,38,39,40,41,42,43,44].

Tchekmedyan et al. enrolled 33 patients with advanced ACC in a phase II clinical trial and used Lenvatinib 24 mg/d. As a result, 15.6% of patients had a partial response (PR), 75% experienced stable disease (SD), 6.3% discontinued treatment due to toxicity, and 3.1% had progression of disease (PD). Median PFS time was 17.5 months and Disease Control Rate was 80.6%.

The most common grade 3 or 4 adverse events were hypertension and oral pain, with a frequence of about 10% of patients. Results of this trial prompted the Regulatory Autorities to approve and register lenvatinib for patients with diagnosis of advanced ACC not suitable for curative surgery and or radiation therapy [38].

Another drug acting on angiogenesis being tested is rivoceranib (apatinib), which inihbits VEGFR-2. A prospective phase 2 clinical trial of this agent was carried out in patients with diagnosis of advanced AdCC. Sixty-five (65) patients were enrolled in this trial and as results, the ORR was 46% and the DCR was 98%, with a median 1-year OS of 92% [39].

The PI3K/Akt/mTHOR pathway is often a downstream effector activated by several of the abovementioned oncoproteins (MYB, NOTCH, various Tirosine Kinase Receptors etc.). Drugs targeting directly PI3K are available in clinical trials, and one of these trial regards AdCC. Hoover et al. enrolled 15 patients with recurrent/metastatic ACC in a phase II trial and treated them with nelfinavir.

Nelfinavir is a drug acting as protease inhibitor, normally employed for treatment of patients with HIV infection. Another mechanism of action of nelfinafir consists of blocking PI3K. Among 15 trial participants, no patients achieved RP or RC, while median PFS in those obtaining SD was 5.5 months [45].

In conclusion, prognosis in patients with ACC remains poor, especially in advanced disease. Systemic therapy can also rely on targeted therapy, and on Lenvatinib in particular. Targeting angiogenesis and targeting VEGFR-stimulated pathways has proven to be a good option for AdCC. The best ORR and Survival Rate have been reached using drugs that target angiogenesis (such as lenvatinib, axitinib and rivoceranib). The results of clinical trials demonstrated that targeting the most frequently disrupted pathway, namely the MYB-stimulated pathway, does not translate in a significant clinical benefit. A more accurate study of mutational status of ACC, and/or a better knowledge of interactions between the disrupted intracellular pathways, could in the near future help in designing more effective targeted strategies.

## 3. Salivary Duct Carcinoma (SDC)

SDCs often arise from major salivary glands and the most frequent site is the parotid gland. SDC accounts for 5% of all SGCs. These tumors may be quite different in terms of aggressiveness, depending on the initial staging. Median OS ranges from 6 months in patients with advanced disease to 50–70 months in those presenting with a locoregional disease [46]. SDC tends to spread from the origin organ early, with lymph nodal metastases seen in 40–70% of cases (at diagnosis) and distant metastases in 5–10% of subjects [47]. Surgery followed or not by adjuvant radiation therapy represents the mainstay of treatment in early and locally advanced disease. Oligometastatic disease may yet benefit from resection of the primary tumor and metastases, but very advanced or multi-metastatic disease is commonly treated with chemotherapy. Platinum-based regimens are the most reported in case studies. The employed schemes are those including carboplatin plus paclitaxel, able to reach an ORR of 40%; another combination is carboplatin plus docetaxel which reaches an ORR of 50%. Other schemes are the same employed for AdCC and in particular the doublet cisplatin-5FU, cisplatin-gemcitabine, and CAP in some reports [48,49,50].

The mutational status of SDC is characterized by some particular DNA changes, specifically HER2 gene amplification, overexpression of Androgen Receptors (ARs), the over-expression of Epidermal Growth Factor Receptors (EGFR), mutations of TP53, mutations of PI3KCA, and mutations of H-RAS [51].

Upregulation of HER2 is found in 30–50% of SDC and it is related to gene amplification. This genic change often occurs in SDC, but other histologies are also involved, such as Adenocarcinoma NOS [52]. HER2 is a member of erythroblastosis oncogene B (ErbB) family, which also comprises three other structurally similar receptors, specifically EGFR, HER3, and HER4. All members of the ErbB family have an extracellular domain (which interacts with the ligand), a hydrophobic transmembrane domain (which anchors itself to the cell membrane), and cytoplasmic tyrosine kinase-containing domain (which transduces the signal in the cell). Each ErbB receptor is not able to function alone, but needs to dimerize with another subunit. With regard to other ERbB receptors, HER2 does not have a specific direct ligand, thus, it acts as a co-receptor and increases the affinity of ligand binding to the dimeric receptor complex. The main heterodimer, responsible for the transduction of the cell-proliferation signal, is that composed of HER2 and EGFR [53].

**Figure 3:**
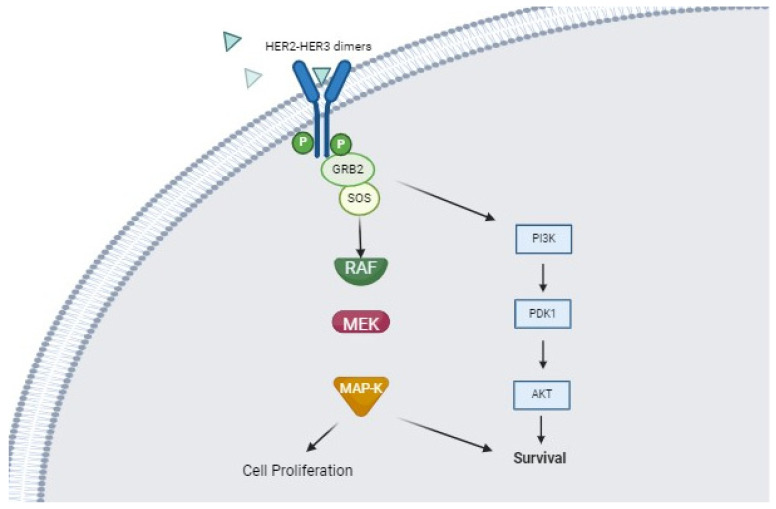
HER2-HER3 are the dimers that are mainly able to stimulate both MAP-K and PI3K mediated pathways, which results in cell survival (immortalization) and proliferation. This may be the most important mechanism able to promote carcinogenesis in HER2 positive SDCs.

ErbB activation promotes cell growth through different intracellular pathways, among which RAS/RAF/MEK/ERK pathway and the PI3K/Akt/mTHOR pathway [53,54]. Figure 3 illustrates the HER2 stimulated pathway, which is able to promote cell proliferation and cell immortalization.

For patients with HER2-positive SGCs (i.e., SDC and Adenocarcinoma NOS), HER2-targeted therapies have been employed with the rationale of blocking the cell proliferation signal.

Takahashi et al. designed a phase II clinical trial enrolling 27 patients with diagnosis of advanced SDC and treated them with the combination of docetaxel and trastuzumab (a monoclonal antibody directed against HER2). The primary endpoint was ORR and, as a result, the response rate reached 71% at a price of a manageable toxicity profile (14% of G3 neutropenia) [55].

Similarly, Lee et al. carried out a phase II study on 43 patients diagnosed with advanced SDC and treated with the same combination docetaxel plus trastuzumab. ORR was 70% and interestingly, the DCR (disease control rate) was 93%. Nevertheless, the rate of permanent therapy discontinuation due to side effects was 30% [56].

Among other HER2-targeted therapies, pertuzumab is a monoclonal antibody whose action mechanism is complementary to trastuzumab, inhibiting ligand-dependent HER2–HER3 dimerization and reducing signaling via intracellular pathways such as phosphatidylinositol 3-kinase (PI3K/Akt) [57]. The combination of pertuzumab and trastuzumab is so able to block not only HER2-HER3 heterodimers, but also EGFR/HER2 ones, resulting in a wider inhibition of the ErbB-stimulated intracellular pathways.

Furthermore, TDM-1 is an antibody-drug conjugate consisting of the humanized monoclonal antibody trastuzumab covalently linked to the cytotoxic agent DM1. It is normally employed in advanced HER2 positive breast cancer. Both pertuzumab and TDM-1 have demonstrated activity and efficacy in HER2 positive SDCs in a clinical report [58].

Uijen et al. analyzed the outcome of 13 advanced SDC patients, who were treated with the combination of trastuzumab, pertuzumab, and docetaxel as the first line treatment, and subsequently trastuzumab emtansine (T-DM1) for second line treatment. In twelve evaluable patients, the authors observed one CR and six PR (ORR 58%), with a median PFS of 6.9 months. Seven patients received subsequent T-DM1 as the second line treatment, obtaining an ORR of 57%, with a median PFS of 4.4 months. The toxicity profile was manageable [58].

**Figure 4:**
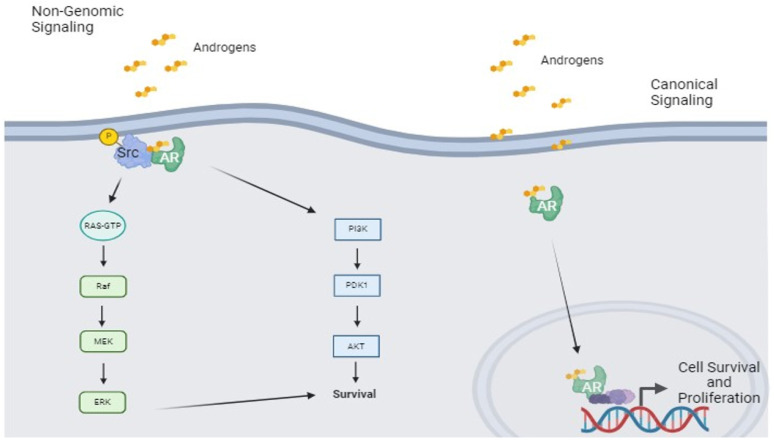
In the above figure are depicted two different routes to obtain the same result (cell proliferation and survival). The over-activation of both canonical and non-genomic signaling are considered to be “driver alterations” in the carcinogenesis of SDC overexpressing AR. AR: Androgen Receptor.

Another important target in the context of SDC (and ADC NOS) is the Androgen Receptor (AR), which is a nuclear steroid hormone receptor able to bind both testosterone and 5α-dihydrotestosterone. It regulates the transcription of several effector genes through direct DNA interactions [59]. Similarly to HER2, AR is often over-expressed in SDCs, and this results in an increase of cell growth potential. AR is primarily located in the cytoplasm; once bound its ligand (testosterone or 5α-dihydrotestosterone), AR forms a homodimer, undergoes a conformational change, and interacts with additional proteins that facilitate its nuclear translocation. In the nucleus, it interacts with the androgen response elements (AREs) on promoter regions, and as result, it enhances the transcription of several genes involved in the cell cycle progression. This abovementioned pathway is called “genomic signaling”. Another way to stimulate cell signaling has also been described and its name is “non-genomic signaling”. The latter is mediated by cytoplasmic AR, which facilitates the activation of kinase-signaling cascades, including the Ras-Raf-1, (PI3K)/Akt and protein kinase C (PKC), which, in turn, converge on the mitogen-activated protein kinase (MAPK) pathway, leading in this way to cell proliferation [60,61]. Figure 4 illustrates the AR mediated intracellular pathway, highlighting its importance in SDC carcinogenesis.

AR is over-expressed in about 65–98% of SDC and this molecular feature has paved the way to clinical trials employing anti-androgen drugs or hormonal therapies acting on the GnRH/LH/testosterone axis [62]. Different drugs inhibiting the AR-mediated pathways have been tested in SDCc and in AR-overexpressing SGCs, with encouraging results which will herein summarize.

In this context, Fushimi et al. conducted a phase II trial enrolling patients with advanced AR-overexpressing SGC and treated them with a combination of Leuprorelin acetate (a GnRH agonist) and Bicalutamide (an anti-androgen agent).

Thirty-six patients were enrolled and most of them (94%) had a diagnosis of SDC. The best ORR was 41.7% and the median PFS and OS were 8.8 and 30.5 months, respectively. The reported toxicity was very low, with 2 patients suffering grade 3 hyper-transaminasemia [63].

Further evidence of the benefits of antiandrogen deprivation therapy (ADT) in AR-positive SGCs was found by Locati et al., who retrospectively analyzed 17 patients with AR-expressing recurrent/metastatic SGC, treated with ADT (Leuprorelin and Bicalutamide). Most of the SGCs were SDC. ORR, the primary endpoint of the study, was 64.7%, while 3-year PFS and 5-year OS were 11.8% and 19.3%, respectively [64].

Despite an initial/moderate response to ADT, AR-expressing SDCs, as well as prostate tumors, also develop resistance and can define castration-resistant tumors. There are many mechanisms underlying resistance to castration and the most important ones are: AR amplification and overexpression, AR splice variants, AR mutations, post-translational regulation of AR, coactivators, or corepressor modification, and intratumoral steroid hormone synthesis.

Some drugs are able to block the proliferative signal stimulated by androgens even in castration-resistant tumors and these drugs are defined as “hyper-castrating agents”. Examples of these latter drugs are abiraterone acetate and enzalutamide [65].

Locati et al. carried out a phase II trial enrolling patients with AR-expressing SGCs treated with abiraterone acetate as second line treatment for those who progressed after ADT. Twenty-four patients with diagnosis of recurrent metastatic castration resistant SGC were enrolled, and the primary endpoint was the ORR. The ORR was 21%, the DCR was 62.5%, and the median duration of response was 5.82 months, with a PFS of 3.65 months and OS of 22.47 months. The G3 side effects were fatigue, flushing, and supraventricular tachycardia, which occurred in 25% of patients [66].

Ho et al. enrolled 46 patients with diagnosis of advanced AR-expressing SGC who progressed after a first line of chemotherapy or ADT. Patients were treated with enzalutamide, a hyper-castrating agent, and the main endpoint of the study was ORR. Twenty-six patients (56.5%) experienced tumor regression in target lesions, The median PFS was 5.6 months and the median OS was 17.0 months. The most common adverse events were fatigue, hypertension, hot flashes, and weight loss [67].

Clinical trials employing targeted therapy for SDC were reported in Table 2.

Based on the abovementioned data, some conclusions can be drawn. First, targeted therapy based on the drugs that act on HER-stimulated pathways has produced good results in terms of activity and efficacy. Trastuzumab, pertuzumab, and TDM-1 have been used in clinical studies, giving good results in patients diagnosed with SDC. However, now none of the aforementioned drugs have received approval from regulatory authorities and reimbursement in clinical practice, except for trastuzumab (in combination with docetaxel) for HER2-overexpressing SDC, according to 648/96 law in Italy.

Conversely, ADT using GnRH analogues and Bicalutamide represents the only specific molecular targeted therapy authorized and reimbursed patients diagnosed with AR-expressing SGC. The aforementioned drugs can be used concomitantly or instead of traditional chemotherapy.

Abiraterone and enzalutamide, despite having demonstrated decent activity and efficacy at the cost of manageable toxicity, have not yet obtained indication and reimbursement in clinical practice, but in the future, they could be used in case of failure of ADT in patients diagnosed with AR-expressing SGC.

## 4. Muco-Epidermoid Carcinoma (MEC)

MEC is the most common histotype among the SGCs, accounting for approximately 20% of all cases and often occurring in the parotid gland, followed by the submandibular and sublingual glands. Prognosis strongly depends on staging of the disease. The 5-year OS ranges from 98% for low grade and localized tumors to 25% for high grade and metastatic MEC [68]. Even for MEC, surgery remains the backbone of treatment in locally advanced/oligometastatic disease. Resection of the primary tumor and of metastatic sites represents the standard of therapy, given its low chemo-sensitivity. High grade/multi-metastatic MEC is treated either with single-agent or polichemotherapy utilizing cisplatin, fluorouracil and paclitaxel. The most employed chemotherapy schemes are combination of cisplatin-vinorelbine, Carboplatin-paclitaxel, Cisplatin-vincristine-5Fluorouracil, cisplatin-gemcitabine, and PAC [69]. Nevertheless, significant objective responses are infrequent and short-lived.

**Figure 5:**
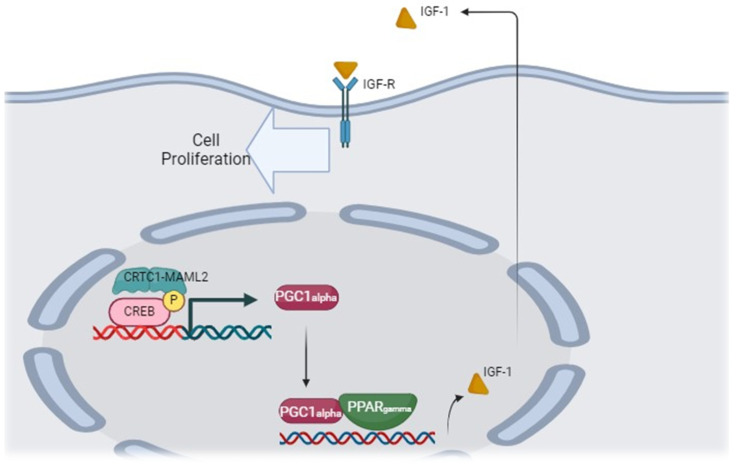
The pathway stimulated by the chimeric protein CRTC1-MAML2 is probably the main pathway responsible for carcinogenesis in MEC.

More than half of MECs have a characteristic genetic alteration, namely t(11;19) (q21–22; p13) translocation leading to a CRTC1-MAML2 fusion gene, which constitutively activates CREB-mediated transcription. As described above for SDCs, HER gene amplification is also present in about 33% of MECs. Other common genetic findings include CDKN2A, TP53, CDKN2B, BAP1, PIK3CA, HRAS, and BRCA alterations [70,71,72]. The mechanism of carcinogenesis induced by CRTC1-MAML chimeric protein is shown in Figure 5.

CRTC1-MAML2 gene fusion has been detected in up to 80% of MEC cases [70] and is currently utilized as a pathognomonic and diagnostic marker of MEC. The chimeric protein named CRTC1-MAML2 potently activates a CREB-dependent transcriptional program with the recruitment of p300/CBP. The latter interacts with the transcriptional activation domain (TAD) of MAML2. MAML2 belongs to the MALM family (MAML1, MAML2, MAML3) and forms a DNA-binding complex with NOTCH proteins, thereby regulating transcriptional events that are specific to the NOTCH pathway, such as cell differentiation and proliferation [73]. The pathway stimulated by chimeric protein CTRC1-MAML2 is depicted in Figure 5.

CRTC1-MAML2 fusion-positive cells have demonstrated in vivo sensitivity to EGFR-targeted therapy in MEC xenograft models [74]. However, there are not yet clinical trials in this context. Although the probable driver mutation in MECs is known (CRTC1-MAML2 fusion), no targeted therapy capable of interacting with this pathway is currently available.

Targeting HER2 in MEC has recently become a significant research interest.

Compared to SDCs, MECs also express AR. The biologic significance of concomitant HER2 and AR expression in MEC remains to be elucidated, although their co-expression is associated with worse clinical outcomes. AR expression has the same importance for MEC shown in SDC [75,76]. MEC overexpressing AR should be treated with androgen deprivation therapy.

Patients diagnosed with MEC have been enrolled in clinical trials using anti-EGFR, but the results shown are poor. The same is valid for clinical trials using sorafenib and cabozantinib, as the subgroup of patients with MEC did not show encouraging data.

We can conclude that, to date, the only standard systemic therapy approved for this histotype is polychemotherapy.

## 5. Secretory Carcinoma (SC)

Previously known as mammary analog secretory carcinoma (MASC), secretory carcinoma (SC) shows immunohistochemical resemblance to secretory carcinoma of the breast. SC group rare and slow-evolving tumors and, although cases metastasizing to the lungs and bones have been described, their prognosis is fair, standing at around 90% at the 5-year stage [77]. Therapeutic strategies use surgery in the majority of cases and treatment may include resection of the primary tumors, as well as of the metastases. This is also the case for SCs in the presence of oligometastatic disease. Even in the case of SCs, systemic treatments are indicated in the presence of multi-metastatic disease that are not candidate to surgery. Even for SCs, polichemiotherapy includes cisplatin-containing doublets (cisplatin-5fluorouracil, cisplatin-docetaxel, cisplatin-doxorubicin, cisplatin-gemcitabine, cisplatin vinorelbine, and carboplatin-paclitaxel) and the CAP schedule [69].

Genetic analysis of SCs has showed a pathognomonic gene alteration that can be defined as a “driver”. Similarly to SC of the breast, almost all salivary SC contain a t(12;15)(p13; q25) translocation leading to the ETV6-NTRK3 fusion gene. The latter is highly specific for the SC histotype. NTRK3 encodes for the Tropomyosin receptor kinase C, a tyrosine kinase growth factor receptor involved in promoting the growth, survival, and cell proliferation [78].

Specifically, NTRK3 belongs to the super-family of Tyrosine Kinase Receptors (TRKs). Once bound neurotrophin-3 (NT3), NTRK3 dymerizes and phosphorylates itself. This phosphorylated intracellular domain serves as docking sites for adaptor proteins that trigger downstream signaling cascades. Some of downstream pathways activated by NTRK3 are Phospholipase C, gamma 1 (PLCgamma1) and PI3K/Akt. Once activated by NTR3, PLCgamma1 catalyzes the formation of inositol 1,4,5-trisphosphate (IP3) and diacylglycerol (DAG) from phosphatidylinositol 4,5-bisphosphate (PIP2). IP3 in turn promotes the release of calcium from the endoplasmic reticulum that activates various calcium regulated intracellular signals related to cell proliferation, migration, and survival. DAG, instead, stimulates very similar pathways, but it acts through the activation of Protein Kinase C (PKC) [79].

**Figure 6:**
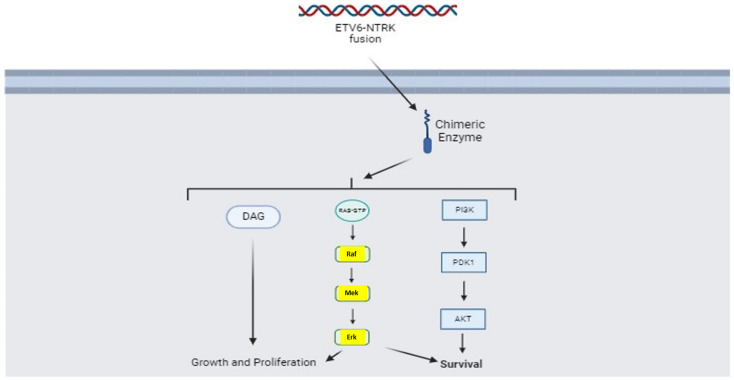
The chimeric protein which results by the ETV6-NTRK fusion gene is able to strongly promote cell survival, growth, and proliferation, acting upon several intracellular pathways.

The ETV6-NTRK3 gene fusion creates a chimeric protein formed by the 5′ end of ETV6 fused to the 3′ end of NTRK3. The ETV6 gene encodes for a transcriptional factor which, when translocated near the NTRK3 gene, causes its constitutive activation with consequent hyper-stimulation of pathways connected to cell proliferation, migration, and survival. The presence of a constitutively active chimeric protein (the ETV6-NTRK3 kinase) means that it can be used as a target for small molecules capable of inhibiting NTRK, such as larotrectinib and entrectinib [80]. Figure 6 shows the abovementioned mechanism of action (NTRK mediated), which may be considered the main promoter of carcinogenesis in SC and all solid tumors bearing ETV6-NTRK fusion.

Drilon et al. enrolled 55 patients with TRK fusion-positive solid-tumors in a phase II trial and treated them with larotrectinib. The ORR was 75% according to independent review and 80% according to investigator assessment. Interestingly, larotrectinib yielded an ORR of 80% among the subgroup of 12 patients with a diagnosis of SC. Side effects were mainly grade 1, and no adverse grade 3/4 events were related to larotrectinib [81].

Hong et al. performed a pooled meta-analysis intended to explore the efficacy and long-term safety of larotrectinib in a larger population of patients with TRK fusion-positive solid tumors. Phase I and II clinical trials enrolling patients with TRK fusion-positive solid tumors were taken into account, and data regarding 159 patients were analyzed. Twenty-one (21) out of 159 patients had a diagnosis of SC. Observed ORR was 79% and grade 3 toxicity, occurring in only 5% of patients, consisted of anemia, neutropenia, and increased alanine aminotransferase. In the subgroup of patients with SC, ORR was instead 90% (19/21), confirming the good results obtained in the registrative trial. Entrectinib has also shown comparable efficacy in clinical trials and, thus, both drugs can be employed in the clinical practice in patients with SGC harboring NTRK-fusion [82].

As SCs belong to the category of tumors in which NTRK fusion is very frequent (approximately 90% of cases), both larotrectinib and entrectinib represent excellent therapeutic possibilities in this category of SGCs (Table 3).

## 6. Immunotherapy for SGCs

Checkpoint inhibitors have also been tested in SGCs. SGCs are extremely heterogeneous from a genetic point of view. TMB is extremely high in SDCs and quite low in ACC. Therefore, it is to be expected that, since the number of tumor neoantigens in SDC is higher, they may be more sensitive to immunotherapy [84,85]. However, TMB does not always correlate with response to immunotherapy. In the presence of an extremely depressed Tumor Microenvironment (TME), it is extremely difficult to elicit an anti-cancer immune response even in the presence of a high number of tumor neo-antigens. Therefore, apart from TMB, other factors also influence the response to immunotherapy. A predictive parameter of response to immunotherapy treatment is certainly the tissue expression of PD-L1. In head and neck squamous cell carcinomas, high PD-L1 expression correlates with a better response to immunotherapy [86]. This last aspect has not been sufficiently studied in SGCs. PD-L1 expression is highly variable among various types of SGCs, ranging from 2 to 71%. The tumors in which this percentage is lower are ACC and Acinic Cell Carcinoma, while the tumors that mostly express PDL-1 are SDC and squamous cell carcinoma [87].

The first small phase Ib study of immunotherapy (with anti-PD-1) in SGCs was the Keynote 028, a basket trial, including 26 patients diagnosed with advanced SGC over-expressing PD-L1. The most common subtype of SGC was adenocarcinoma NOS (38%), followed by MEC (12%), undifferentiated and squamous cell carcinoma (both at 8%), and ACC (8%). Key inclusion criteria included diagnosis of recurrent/metastatic disease, failure of prior systemic therapy and PD-L1 expression ≥ 1% in the tumor tissue. Twenty-six patients with PD-L1 positive SGC were enrolled and treated with pembrolizumab as a single agent. The main endpoint was ORR and after a median follow-up of 20 months, the ORR was 12%, with a median duration of response of 4 months. Side effects were manageable with only 3 patients experiencing grade > 3 toxicity, of which one suffered interstitial pneumonia [88].

On the basis of these poor results, Even et al. carried out a phase II clinical trial enrolling 109 patients with advanced SGCs, regardless of tissue expression of PD-L1. PD-L1 status was defined according to the combined positive score (CPS) and almost 26% of patients enrolled had a PD-L1 positive tumor. From a histological point of view, the majority of patients were diagnosed with ACC (54%). Adenocarcinoma NOS was detected in 23% of cases, while other unspecified histotypes were diagnosed in the remaining 23% of patients. All the patients received at least one dose of Pembrolizumab at dose of 200 mg per square meter of body surface. The ORR in the total population was 4.6%, with one CR and four PRs. In particular, ORR was 10.7% in patients with PD-L1 positive disease and 2.6% in patients with PD-L1 negative disease. The duration of response was 24 months for all 5 responders, median PFS was 4 months, and median OS was 21.1 months. Treatment-related adverse events occurred in 75.2% of patients, the most frequent being fatigue (26.6%), asthenia (14.7%), pruritus (13.8%), and diarrhea (11.9%). Only 15% of patients experienced grade 3–4 toxicity. A very small percentage of patients had high-TMB and notably, 2 out of 3 experienced a response to immunotherapy [89].

Niwa et al. performed a retrospective analysis on 24 patients with a diagnosis of advanced chemo-refractory SGC, who were treated with nivolumab (anti-PD1) as a single agent. The most common histopathological type of cancer was SDC (83%) and 46% of patients had a PD-L1 positive tumor. Only 1 patient out of 24 (4%) experienced an objective response, while two patients with SD maintained the status for more than 24 weeks. Six patients (25%) showed grade 3 or 4 adverse events, and at univariate analysis there was no association between PD-L1 positivity and prognosis [90].

Vos et al. conducted a phase 2 trial evaluating nivolumab and ipilimumab (an anti-CTLA-4) in 64 patients with metastatic SGC. Patients were divided into two histology-based cohorts, specifically those diagnosed with AdCC (cohort 1) and those diagnosed with other histotypes (cohort 2). Both cohorts consisted of 32 patients. From the genetic point of view, 19% of patients in cohort 1 (AdCC) harbored NOTCH1 mutations, while MYB–NFIB gene fusions, putative oncogenic drivers in AdCCs, were found in the majority (60%) of patients. PD-L1 expression on tumor cells was seen in 8% of AdCCs and 23% of non-AdCC SGCs. The primary endpoint (≥4 objective responses) was met in cohort 2 (16%) but not in cohort 1 (6%), highlighting the fact that in AdCCs, which are tumors with low infiltration and lowest TMB, immunotherapy is not effective, while in other SGCs (in particular SDC), the opposite features correlated with better response to check-point inhibitors. Grade 3 toxicity occurred in about 38% of patients, highlighting the worse toxicity profile of Ipilimumab-nivolumab compared to nivolumab alone [91].

Ferrarotto et al. recently conducted a phase II trial evaluating the efficacy of VEGFR inhibitor axitinib and PD-L1 inhibitor avelumab in patients with recurrent/metastatic AdCC. Forty patients enrolled from July 2019 to June 2021; 28 were evaluated for efficacy and activity. The ORR at 6 months was 14% (only PR were seen), the median PFS was 7.3 months, and the median OS was 16.6 months. Most common treatment-related adverse events included fatigue (62%), hypertension (32%), and diarrhea but interestingly, ten (29%) patients had grade 3 adverse events [92].

Immune checkpoint inhibitors do not represent currently standard treatment options in none of SGC histotypes (Table 4).

## 7. Conclusions

SGCs are rare tumors and for this reason few randomized clinical studies are currently available in the scientific literature. Therefore, there is no strong scientific evidence to support one therapy over another.

The prognosis in recurrent/metastatic disease is grim, with anecdotal 5-year survival. Some histotypes, such as AdCC, SC carcinoma, and AciCC, have a better prognosis than others, but the sensitivity to chemotherapy is suboptimal in all histotypes, with the average ORR varying between 20 and 40% [7].

The use of targeted therapy is limited to the AdCC histotype and lenvatinib (TKI inhibitor acting against VEGFR) is the only drug approved and used in clinical practice.

Entrectinib and Larotrectinib (drugs acting against ETV6-NTRK3 kinase) can be used for all malignancies characterized by NTRK-fusion and SCs represent the solid tumor with the highest frequency of the aforementioned mutation.

AR antagonists are commonly used in AR-overexpressing SGCs, the majority of which are SDCs. Trastuzumab could be used in SDCs overexpressing HER-2.

The emergence of translational research in the last 20 years has significantly changed the natural history of some tumors. The identification of particular genetic mutations capable of driving and directing carcinogenesis has led to the development of drugs capable of specifically targeting the altered intracellular pathways. In some cases, this has produced results in clinical practice, but in other cases it has not. In the case of SGCs, several genetic alterations have been identified in the various histotypes and some are typical of a specific histotype. A genetic alteration, however, is considered relevant only if it is itself largely responsible for carcinogenesis and therefore, this mutation is defined as a “driver”.

Notably, it is relevant to identify “driver mutations” in solid tumors, as their targeting is associated with a greater probability of tumor reduction. Unfortunately, the most frequently found mutation in malignancies does not always correspond to the “driver mutation”. For example, the MYB-NFIB fusion, typical of AdCC, does not correspond to the “driver mutation”, since its targeting is not accompanied by a good objective response in clinical trials. The same issue is valid for the CRTC1-MAML2 fusion, which is the main mutation seen in MEC.

Nonetheless, targeting NTRK has changed the natural history of SCs, accompanied by an excellent prognosis, therefore NTRK fusion can be considered a “driver mutation” for SCs.

The “latest generation” immunotherapy, which makes use of checkpoint inhibitors deserves a separate mention. With regard to SGCs, the best results of anti-PD-1 and in some reports of anti-CTLA-4, concern tumors with high TMB and a rich inflammatory lymphocytic infiltrate.

The next step could be the comprehensive study of the tumor genome of SGCs, the identification of frequently disrupted intracellular pathways and their interaction, with the aim of identifying with greater certainty the so-called “weak points” of their carcinogenesis.

## Figures and Tables

**Table 1:** Clinical trials employing targeted therapy for AdCC.

Study	Phase and Drug	Number of pts	ORR %	PFS (Months)	OS (Months)
Pfeffer et al. [30].	*Phase II-Imatinib*	10	00	NR	NR
Ghosal et al. [31].	*Phase II-Imatinib-cDDP*	28	100	15.0	35.0
Hotte et al. [35].	*Phase II-Imatinib*	16	0.05	7.5	NR
Keam et al. [32].	*Phase II-Dovitinib*	32	3.0	6.0	0.0
Dillon et al. [33].	*Phase II-Dovitinib*	35	0.0	8.0	20.5
Chau et al. [34].	*Phase II-Sunitinib*	14	0.05	NR	18.7
Ho et al. [36].	*Phase II-Regorafenib*	38	0.0	NR	NR
Kim et al. [37].	*Phase II-Nintedanib*	20	0.0	NR	NR
Tchekmedyan et al. [38].	*Phase II-Lenvatinib*	33	15.6	17.5	NR
Locati et al. [40].	*Phase II-Lenvatinib*	28	11.5	9.1	27
Locati et al. [41].	*Phase II-Axitinib*	26	8.0	5.5	22
Ho et al. [42].	*Phase II-Axitinib*	33	9.01	5.7	NR
Thomson et al. [43].	*Phase II-Sorafenib*	23	11.0	11.3	19.6
Locati et al. [44].	*Phase II-Sorafenib*	37	16.0	9.0	26.0
Zhu G et al. [39].	*Phase II-Rivoceranib*	65	46.0	NR	NR
Hanna GJ et al. [23].	*Phase II-ATRA*	18	0.0	3.2	NR
Ferrarotto R et al. [25].	*Phase II-AL101*	77	15.4	NR	NR
Hoover et al. [45].	*Phase II-Nelfinavir*	15	0.0	5.5	NR
Ferrarotto et al. [26].	*Phase I-Brontictuzumab*	17	12.0	NR	NR

ORR: overall response rate; PFS: progression free survival; OS: overall survival; ATRA: Retinoic Acid; AL101: gamma secretase inhibitor; NR: not reported.

**Table 2:** Clinical trials employing targeted therapy for SDC.

Study	Phase and Drug	Number of pts	ORR %	PFS (Months)	OS (Months)
Takahashi et al. [55].	*Phase II-Trastuzumab-Docetaxel*	27	71.0	NR	NR
Lee et al. [56].	*Phase II-Trastuzumab-Docetaxel*	43	70.0	NR	NR
Uijen et al. [58].	*Retrospective-trastuzumab, pertuzumab and docetaxel*	13	58.0	6.9	NR
Uijen et al. [58]	*Retrospective–TDM-1*	7	57.0	4.4	NR
Fushimi et al. [63].	*Phase II-Leuprorelin and Bicalutamide*	36	41.7	8.8	30.5
Locati et al. [64].	*Retrospective-Leuprorelin and Bicalutamide*	17	64.7	NR	NR
Locati et al. [66].	*Phase II-Abiraterone*	24	21.0	3.65	22.5
Ho et al. [67].	*Phase II-Enzalutamide*	46	56.0	56	17

ORR: overall response rate; PFS: progression free survival; OS: overall survival; NR: not reported.

**Table 3:** Clinical trials employing targeted therapy for SC.

Study	Phase and Drug	Number of pts	ORR	PFS	OS
Drilon et al. [81].	*Phase II-Larotrectinib*	55 (12 SC)	80%	NR	NR
Hong et al. [82].	*Pooled Meta-Analysis-Larotrectinib*	159 (21 SC)	90%	NR	NR
Doebele et al. [83].	*Pooled Meta-Analysis-Entrectinib*	54 (7 SC)	86%	NR	NR

ORR: overall response rate; PFS: progression free survival; OS: overall survival; NR: not reported.

**Table 4:** Clinical trials employing immunotherapy for SGCs.

Study	Phase and Drug	Number of pts	ORR %	PFS (Months)	OS (Months)
Even et al. [89].	*Phase II-Pembrolizumab*	109	4.6	4	21.1
Cohen et al. [88].	*Phase Ib-Pembrolizumab*	26	12	NR	NR
Niwa et al. [90].	*Retrospective-Nivolumab*	24	4	1.6	10.7
Vos et al. [91].	*Phase II-Ipilimumab + Nivolumab*	64 (32 AdCC)	16 non AdCC 6 AdCC	2.0	NR
Ferrarotto et al. [92].	*Phase II-Avelumab + Axitinib*	40 (AdCC)	14	7.3	16.6

ORR: overall response rate; PFS: progression free survival; OS: overall survival; NR: not reported; ACC: adenoid cystic carcinoma.
